# Curative treatment of oesophageal carcinoma: current options and future developments

**DOI:** 10.1186/1748-717X-6-55

**Published:** 2011-05-26

**Authors:** Maria C Wolf, Michael Stahl, Bernd J Krause, Luigi Bonavina, Christiane Bruns, Claus Belka, Franz Zehentmayr

**Affiliations:** 1 Klinik und Poliklinik für Strahlentherapie und Radioonkologie, Ludwig-Maximilians Universität München, Germany; 2 Klinik für Internistische Onkologie und Hämatologie, Kliniken Essen-Mitte, Germany; 3 Klinik und Poliklinik für Nuklearmedizin, Klinikum rechts der Isar, Technische Universität München, Germany; 4 Klinik und Poliklinik für Nuklearmedizin, Universitätsklinikum Rostock, Germany; 5 Department of Medical and Surgical Sciences, Division of General Surgery, IRCCS, Policlinico San Donato, University of Milan School of Medicine, Milano, Italy; 6 Chirurgische Klinik und Poliklinik, Ludwig-Maximilians Universität München, Germany

## Abstract

Since the 1980s major advances in surgery, radiotherapy and chemotherapy have established multimodal approaches as curative treatment options for oesophageal cancer. In addition the introduction of functional imaging modalities such as PET-CT created new opportunities for a more adequate patient selection and therapy response assessment.

The majority of oesophageal carcinomas are represented by two histologies: squamous cell carcinoma and adenocarcinoma. In recent years an epidemiological shift towards the latter was observed. From a surgical point of view, adenocarcinomas, which are usually located in the distal third of the oesophagus, may be treated with a transhiatal resection, whereas squamous cell carcinomas, which are typically found in the middle and the upper third, require a transthoracic approach. Since overall survival after surgery alone is poor, multimodality approaches have been developed. At least for patients with locally advanced tumors, surgery alone can no longer be advocated as routine treatment. Nowadays, scientific interest is focused on tumor response to induction radiochemotherapy. A neoadjuvant approach includes the early and accurate assessment of clinical response, optimally performed by repeated PET-CT imaging and endoscopic ultrasound, which may permit early adaption of the therapeutic concept. Patients with SCC that show clinical response by PET CT are considered to have a better prognosis, regardless of whether surgery will be performed or not. In non-responding patients salvage surgery improves survival, especially if complete resection is achieved.

## 1. Surgery

In Western countries, the recent epidemiological shift from squamous cell carcinoma to adenocarcinoma arising in Barrett's metaplasia has led to an increasing referral of patients with early oesophageal tumours detected during endoscopic surveillance [1]. Squamous cell carcinoma (SCC) is associated with low socioeconomic status [2], active tobacco and alcohol abuse, malnutrition, liver dysfunction, pulmonary co-morbidities, and second malignancies [3].

Patients with adenocarcinoma (AC) are characterized by co-morbidities such as coronary heart disease and a higher median age [4]. AC is predominantly (94%) located in the lower third of the oesophagus, whereas 51% of SCC are found in the middle third and only 36% in the lower third. Moreover, a better prognosis with a significantly higher overall survival after resection of AC than SCC was reported in some studies [5-7] whereas a SEER database review of 4752 patients showed no difference [8]. However, the majority of patients still present with advanced disease and up to two thirds are inoperable at the time of diagnosis.

Complete resection (R0), N- and T-stage are independent prognostic factors for SCC. Patients are categorised in risk groups by Karnofsky Performance Scale (KPS), cardiac function, liver and lung parameters [9]. Pre-operative improvement of nutritional status, abstention from tobacco and alcohol can decrease the perioperative risk. Patients with SCC of the cervical oesophagus, T1 - 2, with low surgical risk according to Bartels et al. [9], can be treated by a limited resection including regional lymphadenectomy and reconstruction using a free jejunal loop with microsurgical vessel anastomoses, whereas T3-4 patients are treated with neoadjuvant radiochemotherapy. Patients with a high perioperative risk get definitive radiochemotherapy regardless of T-stage. In the low risk situation, T1-2 tumours located in the middle and lower third of the oesophagus are treated with transthoracic en-bloc-oesophagectomy with two-field lymphadenectomy and reconstruction with a gastric tube. Use of the colon as an esophageal substitute is reserved to patients with previous gastric resection. In patients with T3-4 tumours the same surgical strategy is chosen, if possible after preoperative radiochemotherapy. Again, for patients with higher perioperative risk definitive radiochemotherapy is the treatment of choice. For AC R0, T- and N-stage are also independent prognostic markers. Grading is more advantageous in carcinoma of the gastro-oesophageal junction (GEJ) I than GEJ II/III, with 80% of intestinal metaplasia (Barrett's oesophagus) being found in GEJ I [6]. The surgical procedure of choice for GEJ I is subtotal oesophagectomy with proximal gastric resection and a two-field lymphadenectomy, whereas GEJ II/III is treated by transhiatal extended gastric resection and oesophagojejunostomy. For early GEJ I-III a transabdominal limited resection of the distal oesophagus and the proximal stomach with interposition of small intestine (Merendino procedure) can be performed. When transthoracic oesophagectomy (TTE) is compared to the transhiatal oesophagectomy (THE) for adenocarcinoma of the mid and distal oesophagus, no significant difference in overall survival can be observed, but a tendency towards better 5-year survival for TTE in GEJ I and better locoregional control with limited lymphnode invasion have been reported [10,11]. Kato et al. showed a significantly higher overall survival in 3-field versus 2-field lymphadenectomy [12], whereas a randomised trial showed no benefit [13]. Cervical lymphadenectomy seems to be useful in carcinomas located in the cervical and upper third of the oesophagus [13,14]. Transhiatal oesophagectomy is indicated in patients with high pulmonary risk since it decreases early morbidity and mortality but has a trend to worse long term survival. With either a 3-field or a 2-field approach 5-year overall survival rates of 20% can be achieved [15]. Hence, oesophagectomy is a complex operation that entails a two or three-field approach depending on the site of tumor, clinical staging, and Karnofsky performance status. Although overall postoperative mortality has decreased to less than 5% in high-volume centers [16], anastomotic and respiratory failures are still frequent [11]. In the past three decades surgery has developed from transhiatal oesophagectomy [17] to video-assisted surgery [18,19]. Laparoscopy has provided the opportunity of minimally invasive surgical staging [20] and gastric mobilisation with D2 lymphadenectomy extended to the lower mediastinal compartment [21,22]. Furthermore, it was shown that hybrid operations combining laparoscopy and right thoracotomy could be advantageous in regards to respiratory function [23]. A three-stage thoracoscopic oesophagectomy with cervical anastomosis may represent a better minimally invasive surgical option in SCC patients [24,25]. Expected advantages of minimal access techniques include a decrease in postoperative pain, inflammatory cytokine production, cardiopulmonary complications, blood loss, and the length of hospital stay. Although short and medium-term efficacy of these procedures have been proven [26-28], results are still inconclusive. As multicentre studies are not available and because of problems with standardization of such complex procedures, the effectiveness of minimal access oesophageal surgery is difficult to demonstrate.

In summary, from a surgical point of view, AC and SCC need separate therapeutic strategies for which accurate patient selection (staging, evaluation of co-morbidities) is indispensable. Minimally invasive oesophageal surgery is evolving and may become increasingly important. Still, it is hard to imagine that the management of oesophageal cancer will merely be based on improved surgery. Instead, surgeons should be ready for a new scenario, which comprises biological tumour staging and targeted therapies combined with neoadjuvant radiochemotherapy.

## 2. Radiochemotherapy

For the past three decades combined modality treatment for cancer of the oesophagus has been investigated in a number of studies with the intention to improve long-term outcome. Because of disappointing results of the intergroup study 0113 [29] perioperative treatment for oesophageal cancer has been a matter of debate for a long time. Nowadays we know that the non-stratified mixture of patients led to a bias. Meanwhile, six meta-analyses show the value of perioperative radiochemotherapy [30-35].

### 2.1 Radiochemotherapy as definitive treatment

One of the first studies analyzing the efficacy of radiochemotherapy as definitive treatment was the RTOG 85-01 trial [36,37], which revealed the superiority of radiochemotherapy compared to radiotherapy alone in regards to 5-years overall survival. Acute toxicity was higher in the combined treatment arm, yet no difference in long term toxicity could be observed. This trial still exerts a major influence in clinical practice. A meta-analysis by Wong including 19 (11 concomitant radiochemotherapy, 8 sequential) trials that compare radiochemotherapy versus radiotherapy alone concludes that concomitant radiochemotherapy is better than sequential radiochemotherapy in regards to overall survival, disease free survival and local control [38]. The only study that compared definitive radiochemotherapy to surgery alone found no statistically significant difference for overall survival and disease free survival [39] showing, that neither of the two treatment modalities is superior. This study was criticized for ethical inadequacies (e.g.: no informed consent) and therefore published only with reserve. Although the intergroup dose escalation study (INT 123) found no benefit for an increase from 50.4 to 64.8 Gy, a moderate dose escalation seems useful [40,41].

### 2.2 Radiochemotherapy in multimodal treatment approaches

Several studies and three metaanalyses showed a statistically significant survival benefit for preoperative radiochemotherapy plus surgery versus surgery alone [31,32,35,42,43]. Fiorica found that the effect of preoperative radiochemotherapy is even more pronounced in patients with adenocarcinoma [35]. A metaanalysis performed by Gebski et al. revealed that both SCC and adenocarcinoma benefit from preoperative radiochemotherapy [31]. The problem with some of these trials is that - by current standards - low to moderate doses were used because of crude methods of radiation planning and delivery at the time. Three other metaanalyses showed no significant survival advantage for preoperative radiochemotherapy [33,34,44]. Due to this inconclusiveness we hypothesize that overall survival alone may be an insufficient parameter to describe the effectiveness of preoperative radiochemotherapy. In an interesting study Berger et al. correlate overall survival with complete pathological response (pCR). The 5-year survival of patients who achieved pCR after preoperative radiochemotherapy was almost 50% [45]. The second independent predictive marker for overall survival was complete resection (R0). Thus, the question arises whether pCR is an integrative biomarker for generally better prognosis or a pre-requisite for more effective surgery, in both cases better outcome can be expected. This is confirmed by two other studies [46,47].

The trials performed by Stahl et al. and Bedenne et al. showed improved local control with radiochemotherapy followed by surgery compared to radiochemotherapy alone. An important result of these studies is that patients with tumour response to induction chemo(radio)therapy constitute a favorable prognostic subgroup. Nevertheless, treatment related mortality in the surgery arm was 12.8% as opposed to 3.5% with radiochemotherapy only [48,49]. These studies suggest that tumour response to induction radiochemotherapy might help to identify patients with good prognosis, regardless of whether surgery will be performed or not. In these patients surgery can no longer be recommended as routine treatment [49,50]. But in the group of non-responders surgery improved survival, especially if complete resection has been achieved. Future studies are warranted to increase the number of responders to induction treatment and to investigate dose escalation regimens. In these studies the integration of functional imaging methods for response evaluation is indispensable.

## 3. PET/CT for staging and response prediction

Endoscopic ultrasound and computer tomography (CT) are primarily used for the assessment of local tumour invasion and locoregional lymph node involvement. For detection of local lymph node metastases, Positron emission tomography (PET) with the glucose analogue 2'-[18F]-fluoro-2'-deoxy-D-glucose (FDG) has a limited sensitivity and specificity of 57% (95% CI, 43%-70%) and 85% (95% CI, 76%-95%), respectively [51]. Therefore, in the detection of locoregional disease, PET appears to be inferior to endoscopic ultrasonography. But for the purpose of M-staging FDG-PET is very useful with a sensitivity and specificity of 71% (95% CI, 62%-79%) and 93% (95% CI, 89%-97%) [51,52], which is crucial for the differentiation between locoregional and systemic disease. In adenocarcinomas of the oesophago-gastric junction (GEJ), FDG has been established and validated as a surrogate marker for therapy response assessment. A number of studies showed that FDG-PET allows prediction of response and prognosis whereas in other studies FDG-PET was not predictive for response and prognosis [53]. The MUNICON trial is a unicentre study, which showed that a PET guided treatment algorithm in patients with adenocarcinoma of the oesophago-gastric junction is feasible [54]. The results of this study are important concerning the individualization of multimodal treatment approaches. The use of FDG PET and PET/CT for therapy monitoring in oesophageal cancer is the subject of intense discussion, underlining the need for randomized multicentre studies.

## 4. Summary

In summary, the following therapeutic strategies can be proposed: surgical resection for stage I and IIA, neoadjuvant chemotherapy (adenocarcinomas) or radiochemotherapy (squamous cell or adenocarcinomas) plus surgery for stage IIB. In locally advanced oesophageal cancer (stage III) - if surgery is potentially possible - neoadjuvant radiochemotherapy should be followed by surgery in patients with adenocarcinomas or those patients with SCC without morphological response after chemo(radio)therapy. For responders with SCC we consider completion of radiochemotherapy to be the most appropriate treatment option. Future tasks comprise improved delivery of radiochemotherapy by integration of techniques such as IMRT to reduce toxicity, a better understanding of tumour response by research on molecular profiles to predict pCR and finally clinical evaluation of neoadjuvant treatment by PET-CT imaging combined with endoscopic ultrasound [50].

## Competing interests

The authors declare that they have no competing interests.

## Authors' contributions

MCW, CB1, LB: wrote and compiled the chapter surgery, responsible for content. MCW, MS, CB2, FZ wrote and compiled the chapter radiochemotherapy, responsible for content. BJK: wrote the chapter PET/CT, responsible for content. MCW, FZ: wrote the abstract and the summary, responsible for content. MCW, FZ, CB2: participated at the revision. All authors read and approved the final manuscript.
